# The need for nursing instruction in patients receiving steroid pulse therapy for the treatment of autoimmune diseases and the effect of instruction on patient knowledge

**DOI:** 10.1186/1471-2474-11-217

**Published:** 2010-09-21

**Authors:** Yu-Chu Pai

**Affiliations:** 1 Supervisor, Nursing Department, Taipei Veterans General Hospital, Taiwan

## Abstract

**Background:**

Many patients who receive steroid pulse therapy go home the same day or the day after steroid administration. Nursing instructions are important for improving patient knowledge related to their diseases and treatments, but the short hospital stay often prevents complete education and guidance regarding the given therapy. The purpose of this study was to investigate the need for nursing instruction in patients receiving steroid pulse therapy for the treatment of autoimmune diseases and the effect of instruction on patient knowledge of their disease and treatment.

**Methods:**

Patients with systemic lupus erythematosus (SLE) and systemic sclerosis receiving steroid pulse therapy (N = 63) were recruited from a medical center in Taipei. A structured questionnaire was used for data collection before and after nursing instruction, and 1 week as well as 2 weeks after therapy. The need for nursing instruction and knowledge levels were validated using Cronbach's α reliability test.

**Results:**

There was a significant difference (*P *< 0.001) in the need for nursing instruction among the 4 time points. There was a positive correlation between the need for nursing instruction and body weight change, frequency of treatment, and distress, but there was a negative correlation with knowledge level (β = -0.012, *P *= 0.003) regarding symptoms. The knowledge level of subjects after nursing instruction was significantly higher than before nursing instruction (80 ± 14.31 vs. 70.06 ± 17.23, *P *< 0.001).

**Conclusions:**

This study indicates that nursing instruction is needed by patients receiving steroid pulse therapy, and that by designing and administering nursing instructions according to the priority of patient symptoms, nurses can improve patient knowledge related to their diseases and treatments. In addition, the need for nursing instruction can be affected by patient characteristics.

## Background

In medicine and nursing, care workers have to understand their patients in relation to their concerns and the experience of hospitalization, and fulfill their physical and psychological needs. A recent report indicated that the 2 most important concerns of inpatients were their physical condition and effective nursing care [1]. Effective, quality care is the ability to assess the patient's ability and provide him/her appropriate education to assist them in self-care [2], which can lead to a reduction in medical costs [3].

Due to changes in health care and the impact of rising medical costs, hospital stays have become shorter, challenging nurses to provide inpatients with complete and adequate education and guidance. Nursing instructions are an important factor influencing patient perceptions, and before drafting instructions, nurses must understand a patient's needs and factors affecting those needs [4]. Nursing instruction should be patient-centered and coupled with a patient's knowledge and education level in order to provide the appropriate information to improve their self-care ability as well as knowledge level.

Steroid pulse therapy has been applied to patients with rheumatic or autoimmune diseases since the 1970 s. Large doses of immunosuppressive agents are administered to delay disease progression and reduce physical discomforts. Associated symptoms such as myalgia, arthralgia, gastrointestinal upset, and neurological effects (e.g., sleep disturbance, headaches) may lower a patient's willingness to undergo steroid pulse therapy [5]. Patients with autoimmune diseases undergoing steroid pulse therapy typically undergo repetitive treatments and hospital admissions. With steroid pulse therapy, a relatively high dose of steroid is administrated for 3 days. If the symptoms are under control after the 3 days of treatment, the patients will typically be discharged immediately or shortly after the treatment is completed. If the symptom persists after 3 days of treatment, the patient will be kept in the hospital while oral steroids are administered and until their condition has stabilized. Difficulties can arise for patient home care secondary to lack of instruction and guidance from the clinical team [6,7]. Patient knowledge and understanding of common side effects and symptoms associated with steroid pulse therapy is important, and systematic nursing procedures and instructions provide patients and their families with relevant information to diminish the anxiety associated with the disease and treatment, promote compliance with management, and to enhance patient self-care ability, thus reducing the occurrence of home care problems [8]. Education of these patients may potentially reduce symptoms associated with therapy and improve the health and quality of life of the patients.

The aim of this study was to investigate the need for nursing instruction in patients receiving steroid pulse therapy for the treatment of autoimmune diseases and the effect of instruction on patient knowledge of their disease and treatment.

## Methods

**Figure 1:**
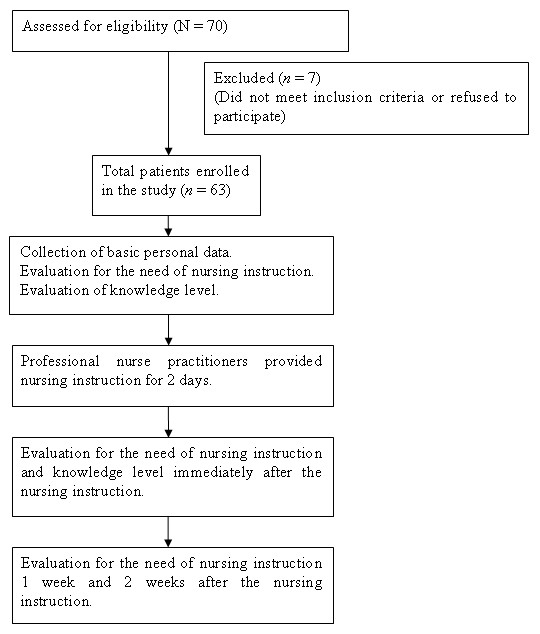
**Flow chart of the study design**.

This descriptive, comparative research study was performed in the Divisions of Allergy, Immunology, and Rheumatology of a medical center in Taipei City, Taiwan. Subjects were a convenience sample of patients receiving steroid pulse therapy, and those who consented to participate in the study were surveyed by means of a structured questionnaire to collect data. Patients were queried before beginning steroid pulse therapy and followed until 2 weeks after completion of therapy (Figure 1). All tested patients had clear consciousness, no psychological abnormalities, and good verbal and written communication abilities. Before data collection, the purpose of the study was explained to the patients, and the written informed consent was obtained from each patient. This study was conducted in accordance with Good Clinical Practice and the Declaration of Helsinki and its amendments. The protocol was approved by the Institutional Review Board of Taipei Veterans General Hospital (Reference No. 94-03-17A).

The study instrument included basic personal data such as age, gender, body weight, education level, therapy dosage, the number of times they have received therapy, diagnosis, and length of hospital admission. The need of nursing instruction described patients' needs in order that they can understand the diseases and therapy procedures, effects and side effects of drugs, management of side effects, diet restrictions, significance of each test, daily care, and methods of emergency medical treatment. The need of nursing instruction was measured once before steroid pulse therapy. Then, professional nurse practitioners provided nursing instruction for 2 days. The nursing instruction administered was standardized, both verbal and written, individualized to the patient, and was provided for a minimum of 10 minutes each day. The need of nursing instruction was then evaluated immediately after instruction, and at 1 week and 2 weeks after therapy. The evaluation sheet for the need of nursing instruction included 45 questions divided into 9 categories, 5 questions in each category. The 9 categories consisted of questions related to the cardio-pulmonary, endocrine, musculoskeletal, digestive, nervous, optic, integument, immune, and reproductive systems. The evaluation was conducted using a 5-point Likert scale; subjects answered every question in the survey according to the degree of their need of nursing instruction, i.e., whether the patient required instruction with respect to the specific system. A higher score reflected a greater need, i.e., 5 = large need, 1 = little or no need.

To evaluate knowledge level before and after nursing instruction, we examined related references [9] and consulted with 5 experienced clinical nursing experts in order to draft an evaluation instrument. The questionnaire for evaluating knowledge level included understanding of diseases and therapy procedures, effects and side effects of drugs, management of side effects, diet restrictions, significance of each test, recommendations for daily care, and methods of emergency medical treatment. Ten yes/no questions and 10 multiple-choice questions were designed. Each question was valued at 5 points; thus, a total of 100 points for 20 questions. Results were collected before and after nursing instruction and the score difference between two trials was analyzed.

The level of symptom distress was measured with the Symptom Distress Scale-Chinese Modified Form (SDS-CMF), that was original developed and validated by Lai [10] with a Cronbach's alpha of 0.85 and an expert agreement of > 80% for validity. It is a modified form of the Symptom Distress Scale originally developed by McCorkle and Young [11]. The modified SDS-CMF in this study consisted of symptoms associated with pulse therapy in the 9 categories described above. The instrument is self-reported, and rated on a 4-point Likert scale with 1 representing "no symptom" or "no distress," and 4 representing "severe distress." Data were expressed as the mean score ± SD of five items for each category.

The need for nursing instruction and knowledge level questionnaires and the modified SDS-CMF were validated by 5 experts from the related fields of allergy, immunology, and rheumatology based on the level of importance, clarity, and relevance of the content. The evaluation was conducted using a 5-point Likert scale. Items rating below 3 points were removed. The content validity index (CVI) was 0.84. The coefficients of variability determined by Cronbach's α values were 0.966 for the need of nursing instruction questionnaire, 0.896 for knowledge level questionnaire, and 0.812 for the modified SDS-CMF, thus, a high degree consistency was achieved.

### Statistical Analysis

A sample size calculation was performed with a sample size calculator by Rasoft, Inc. http://www.raosoft.com/samplesize.html. Sample size was calculated based on the following formulas where the sample size "n" and margin of error "E" are given by: x = Z(c/100)^2^r(100-r); n = N x/((N-1)E^2 ^+ x); and E = Sqrt[(N - n)x/n(N-1)], where N is the population size, r is the fraction of responses that you are interested in, and Z(c/100) is the critical value for the confidence level, c. In this study, we set the margin of error to 5%, confidence level to 95%, assumed a population size of 75, and a response rate of 50%; thus, recommended sample size was 63.

In general analysis, data were expressed as mean ± standard deviation (SD) for continuous variables, and number with percentage for categorical variables. The Likert 5-point scale was used to evaluate the need of nursing instruction, and the knowledge level of patients. To assess the difference among levels of nursing instruction related factors (age, gender, education levels, body weight change, drug levels, and frequency of treatment, diagnosis, and length of stay in hospital), a paired *t*-test or one-way ANOVA with a pair-wise post-hoc test were performed. Moreover, for detecting the change in need of nursing instruction at the different time points, a general mixed model was performed to select the related factors. A *P *value <0.05 was used to indicate statistical significance. All statistical analyses were performed using SPSS 15.0 (SPSS Inc, Chicago, IL, USA).

## Results

A total of 63 subjects receiving steroid pulse therapy with methylprednisolone (Pfizer, NY, USA) were enrolled and completed the surveys. Patient demographic data and characteristics are presented in Table 1. The majority of subjects were female (49, 77.8%). There were 26 subjects (41.2%) under 30 years of age, 20 subjects (31.8%) above 40 years of age, and 17 subjects (27.0%) between 31 and 40 years of age. There were 37 subjects (58.7%) who received a dose of methylprednisolone >500 mg, and 26 subjects (41.3%) who received ≤500 mg. There were 34 subjects (54%) who had received treatment two times or more, and 29 subjects who had received treatment one time. Among the 63 subjects, there were 43 (68.2%) diagnosed with systemic lupus erythematosus (SLE). Therefore, we categorized those 43 patients into a "SLE group". The other 20 patients had systemic sclerosis.

**Table 1:** Demographics data and characteristics of study subjects (N = 63)

Variable	Number (%)
Age (years)	
≤30	26 (41.2)
31-40	17 (27.0)
> 40	20 (31.8)
Gender	
Male	14 (22.2)
Female	49 (77.8)
Education level	
Below college	32 (50.8)
College and above	31 (49.2)
Body weight change (kg)	
< 2	49 (77.8)
≥2	14 (22.2)
Drug dosage (mg)*	
≤500	26 (41.3)
> 501	37 (58.7)
Frequency of treatment	
1	29 (46.0)
≥2	34 (54.0)
Diagnosis^†^	
SLE group	43 (68.2)
Non-SLE group	20 (31.8)
Hospital stay (days)	
≤5	20 (31.8)
6-10	20 (31.8)
≥11	23 (36.4)

*Patients receive methylprednisolone treatment.

^†^Among the 63 subjects, there were 43 diagnosed with systemic lupus erythematosus (SLE group). Among those patients, 4 were complicated with RA and 6 were complicated with sicca syndrome. The other 20 patients had systemic sclerosis (non-SLE group).

The relationship between patient characteristics and the level of symptom distress, i.e., concern over symptoms related to the particular category, associated with pulse therapy in the 9 categories is presented in Table 2. In general, the level of symptom distress varied significantly with levels of body weight increase and length of hospital stay. In males, the level of symptom distress was higher than females for symptoms associated with the musculoskeletal, nervous, and optic systems, but lower than females for symptoms associated with the reproductive system. Patients with a body weight change >2 kg had a higher level of symptom distress associated with the cardio-pulmonary, endocrine, musculoskeletal, gastrointestinal, and optic systems than patients with a weight change ≤2 kg. Patients with SLE had a higher level of symptom distress associated with the musculoskeletal system than patients without SLE. Patients with a length of hospital stay >5 days had a higher level of symptom distress associated with the nervous system than patients with a shorter length of stay.

**Table 2:** Relationship between patient characteristics and level of symptom distress before intervention associated with pulse therapy (N = 63)

	Cardio-pulmonary		Endocrine		Musculoskeletal		Gastrointestinal		Nervous		Optic		Integument		Immune		Reproductive		Overall	
Variable		*P*		*P*		*P*		*P*		*P*		*P*		*P*		*P*		*P*		*P*
Age (years)																				
≤30	2.24 ± 0.85		1.84 ± 0.65		2.35 ± 1.23		2.11 ± 0.84		1.93 ± 1.03		1.37 ± 0.49		2.65 ± 1.27		1.74 ± 0.80		1.44 ± 0.53		1.94 ± 0.60	
31-40	1.95 ± 0.76		1.86 ± 0.74		2.38 ± 1.30		2.05 ± 1.08		1.89 ± 0.83		1.48 ± 0.97		2.06 ± 1.12		1.52 ± 0.76		1.47 ± 0.75		1.84 ± 0.71	
> 40	2.10 ± 0.76	0.518	1.77 ± 0.66	0.912	2.34 ± 1.21	0.996	1.97 ± 0.83	0.878	2.17 ± 0.85	0.591	2.52 ± 1.14	< 0.001*	2.10 ± 1.04	0.172	1.71 ± 0.63	0.609	1.24 ± 0.51	0.418	1.97 ± 0.55	0.790
																				
Gender																				
Male	2.13 ± 0.61		2.10 ± 0.63		3.00 ± 1.37		2.39 ± 0.89		2.41 ± 0.91		2.29 ± 1.59		2.27 ± 1.30		1.87 ± 0.73		1.09 ± 0.18		2.15 ± 0.64	
Female	2.11 ± 0.84	0.953	1.74 ± 0.66	0.076	2.17 ± 1.17	0.023*	1.95 ± 0.88	0.109	1.88 ± 0.89	0.053	1.62 ± 0.72	0.027*	2.33 ± 1.15	0.878	1.61 ± 0.73	0.248	1.47 ± 0.64	0.031*	1.86 ± 0.59	0.122
																				
Education level																				
Below college	2.14 ± 0.86		1.83 ± 0.67		2.34 ± 1.26		1.97 ± 0.81		1.96 ± 0.93		1.79 ± 0.91		2.39 ± 1.28		1.76 ± 0.78		1.38 ± 0.49		1.93 ± 0.63	
College and above	2.09 ± 0.73	0.792	1.81 ± 0.67	0.914	2.37 ± 1.21	0.923	2.13 ± 0.98	0.481	2.04 ± 0.92	0.725	1.74 ± 01.11	0.820	2.24 ± 1.08	0.620	1.57 ± 0.68	0.313	1.39 ± 0.68	0.902	1.92 ± 0.59	0.927
																				
Body weight change (kg)																				
< 2	1.98 ± 0.73		1.71 ± 0.65		2.11 ± 1.09		1.89 ± 0.79		1.88 ± 0.85		1.62 ± 0.85		2.24 ± 1.17		1.64 ± 0.74		1.39 ± 0.61		1.82 ± 0.57	
≥2	2.60 ± 0.86	0.008*	2.21 ± 0.59	0.011*	3.19 ± 1.32	0.003*	2.61 ± 1.02	0.006*	2.41 ± 1.05	0.053	2.26 ± 1.36	0.037*	2.26 ± 1.21	0.386	1.77 ± 0.73	0.562	1.36 ± 0.52	0.848	2.30 ± 0.61	0.007*
																				
Drug dosage (mg)																				
≤500	2.09 ± 0.86		1.85 ± 0.66		2.18 ± 1.09		2.04 ± 1.01		1.84 ± 0.89		1.48 ± 0.60		2.55 ± 1.25		1.64 ± 0.74		1.39 ± 0.57		1.88 ± 0.62	
> 501	2.14 ± 0.75	0.835	1.81 ± 0.68	0.813	2.48 ± 1.32	0.344	2.05 ± 0.82	0.940	2.11 ± 0.93	0.255	1.96 ± 1.18	0.063	2.15 ± 1.11	0.192	1.69 ± 0.74	0.779	1.38 ± 0.61	0.928	1.96 ± 0.61	0.627
																				
Frequency of treatment																				
1	1.97 ± 0.75		1.72 ± 0.61		2.43 ± 1.33		1.89 ± 1.01		2.10 ± 0.97		1.58 ± 0.92		2.08 ± 1.56		1.56 ± 0.66		1.28 ± 0.48		1.83 ± 0.59	
≥2	2.24 ± 0.82	0.182	1.91 ± 0.71	0.251	2.29 ± 1.14	0.660	2.18 ± 0.77	0.197	1.91 ± 0.88	0.431	1.92 ± 1.07	0.178	2.51 ± 1.17	0.151	1.76 ± 0.79	0.271	1.48 ± 0.67	0.181	2.01 ± 0.62	0.246
																				
Diagnosis																				
SLE group	1.95 ± 0.74		1.75 ± 0.60		2.87 ± 1.49		2.12 ± 0.99		2.08 ± 0.93		1.66 ± 1.86		2.04 ± 1.28		1.59 ± 0.697		1.16 ± 0.30		1.89 ± 0.60	
Non-SLE group	2.19 ± 0.81	0.256	1.86 ± 0.70	0.561	2.11 ± 1.01	0.021*	2.01 ± 0.85	0.665	1.96 ± 0.92	0.628	1.81 ± 0.98	0.576	2.44 ± 1.12	0.210	1.71 ± 0.756	0.561	1.49 ± 0.66	0.039*	1.94 ± 0.62	0.790
																				
Hospital stay (days)																				
≤5	1.82 ± 0.61		1.56 ± 0.55		1.93 ± 1.15		1.67 ± 0.49		1.53 ± 0.57		1.46 ± 0.69		1.98 ± 1.06		1.65 ± 00.70		1.24 ± 00.42		1.63 ± 0.42	
6-10	2.21 ± 0.81		1.93 ± 0.59		2.60 ± 1.24		2.17 ± 1.06		2.22 ± 0.91		1.97 ± 1.16		2.44 ± 1.03		1.62 ± 00.73		1.44 ± 0.69		2.04 ± 0.598	
≥11	2.30 ± 0.87	0.119	1.96 ± 0.77	0.101	2.50 ± 1.23	0.170	2.27 ± 0.94	0.066	2.21 ± 1.05	0.020*	1.85 ± 1.07	0.245	2.49 ± 1.37	0.308	1.73 ± 00.79	0.880	1.46 ± 0.63	0.422	2.07 ± 0.68	0.034*

*P < 0.05.

Data were expressed as mean ± SD

*P *value derived from ANOVA test, α = 0.05.

The relationship between patient characteristics and the need of nursing instruction at 4 time points associated with pulse therapy is shown in Table 3. The need of nursing instruction overall was an average of 1.95 ± 0.99 before pulse therapy, and then decreased to 1.82 ± 0.93 (*P *= 0.03) immediately after pulse therapy, 0.40 ± 0.16 (*P *< 0.001) one week after pulse therapy, and 1.34 ± 0.44 (*P *< 0.001) at two weeks after pulse therapy.

**Table 3:** Relationship between patient characteristics and need of nursing instruction at four time points during pulse therapy (N = 63)

	Before		Immediately After		One Week After		Two Weeks After	
Variable		*P*		*P*		*P*		*P*
Overall^a^	1.95 ± 0.99	-	1.82 ± 0.93	0.030*	0.40 ± 0.16	< 0.001*	1.34 ± 0.44	< 0.001*
								
Age (years)								
≤30	1.95 ± 0.98		1.71 ± 0.96		0.38 ± 0.14		1.30 ± 0.37	
31-40	1.68 ± 0.99		1.58 ± 0.77		0.39 ± 0.18		1.31 ± 0.48	
> 40	2.18 ± 0.99	0.324	2.16 ± 0.95	0.123	0.44 ± 0.16	0.504	1.43 ± 0.51	0.597
								
Gender								
Male	2.37 ± 1.14		2.00 ± 0.79		0.45 ± 0.17		1.38 ± 0.42	
Female	1.83 ± 0.93	0.075	1.77 ± 0.96	0.401	0.39 ± 0.15	0.193	1.33 ± 0.45	0.700
								
Education level								
Below college	2.07 ± 1.18		2.01 ± 1.12		0.41 ± 0.17		1.37 ± .48	
College and above	1.83 ± 0.76	0.340	1.62 ± 0.64	0.100	0.40 ± 0.15	0.843	1.31 ± 0.41	0.569
								
Body weight change (kg)								
< 2	1.69 ± 0.69		1.61 ± 0.73		0.37 ± 0.14		1.28 ± 0.38	
≥2	2.84 ± 1.36	< 0.001*	2.54 ± 01.19	0.001*	0.50 ± 0.18	0.009	1.57 ± 0.59	0.031*
								
Drug dosage (mg)								
≤500	1.75 ± 0.79		1.65 ± 0.74		0.38 ± 0.15		1.32 ± 0.41	
> 501	2.09 ± 1.10	0.175	1.94 ± 1.03	0.227	0.41 ± 0.17	0.455	1.36 ± 0.47	0.737
								
Frequency of treatment								
1	1.72 ± 0.63		1.57 ± 0.59		0.39 ± 0.14		1.31 ± 0.39	
≥2	2.15 ± 1.19	0.088	2.03 ± 1.11	0.048*	0.41 ± 0.17	0.735	1.36 ± 0.49	0.657
								
Diagnosis								
SLE group	2.14 ± 1.16		2.03 ± 1.09		0.41 ± 0.16		1.29 ± 0.37	
Non-SLE group	1.87 ± 0.91	0.310	1.72 ± 0.84	0.230	0.40 ± 0.16	0.727	1.36 ± 0.48	0.547
								
Hospital stay (days)								
≤5	1.55 ± 0.92		1.55 ± 0.99		0.34 ± 0.13		1.23 ± 0.45	
6-10	2.09 ± 0.91		1.83 ± 0.68		0.38 ± 0.13		1.24 ± 0.27	
≥11	2.19 ± 1.05	0.080	2.04 ± 1.03	0.230	0.47 ± 0.18	0.019*	1.52 ± 0.51	0.043*

Need for nursing instruction was evaluated using a 5-point Likert scale, with 5 indicating the greatest need.

*P < 0.05.

Data were expressed as mean ± SD

^a ^*P *value derived from paired t-test for overall, and ANOVA test among multiple categories of other specific variables, α = 0.05.

Patients with a >2 kg weight change had a significantly higher level of need for nursing instruction than patients with a smaller weight change at all time points. Patients staying in the hospital >5 days had a higher level of need of nursing instruction than patients with shorter stays 1 week after pulse therapy. Patients in the hospital for >10 days had a higher level of need of nursing than patients staying <10 days 2 weeks after pulse therapy.

The results of the mixed model analysis for correlation between the need of nursing instruction and patient characteristics is presented in Table 4. The independent variable was set as "need of nursing instruction overall," and the dependent variables were set as body weight change, frequency of treatment, length of hospital stay, symptom distress, and knowledge level. Results indicated that the level of need of nursing instruction was significantly positively correlated with body weight change (β = 0.460, *P *= 0.005), frequency of treatment (β = 0.327, *P *= 0.019), and symptom distress (β = 0.777, *P *< 0.001), but negatively correlated with knowledge level (β = - 0.012, *P *= 0.003). However, nursing instruction did improve the knowledge level regarding steroid pulse therapy. The knowledge level of subjects after nursing instruction was significantly higher than that of subjects before nursing instruction (80 ± 14.31 vs. 70.06 ± 17.23, *P *< 0.001, paired *t*-test) (Table 5).

**Table 4:** Mixed model analysis of correlation between patient characteristics and the need for nursing instruction

Effect	β	SE	*P*
Intercept	0.980	0.3537	0.007*
Body weight change (kg)			
< 2		.	.
≥2	0.460	0.1617	0.005*
Frequency of treatment			
1		.	.
≥2	0.327	0.137	0.019*
Hospital stay (days)			
≤5		.	.
6-10	0.079	0.1594	0.620
≥11	0.103	0.1703	0.548
Symptom distress	0.777	0.1079	< 0.001*
Knowledge level	-0.012	0.0039	0.003*

*P < 0.05.

**Table 5:** Knowledge level before and after nursing instruction (N = 63)

Variable	Knowledge Score (mean ± SD)	Paired *t*-test	*P*
Before nursing instruction	70.06 **± **17.23	-4.60	< 0.001*
After nursing instruction	80.00 **± **14.31		

*Knowledge level of subjects after nursing instruction was significantly higher than before nursing instruction (*P *< 0.001).

## Discussion

Nursing instruction refers to the use of nursing staff teaching skills to provide patients with planned learning methods, and to subsequently expand patients' health knowledge and influence their self-care behavior. Effective nursing instruction can be achieved through specific teaching skills and methods and the use of teaching aids such as slides, manuals, and videotapes. Utilization of well-planned nursing instruction has been a critical aspect of nursing activity. Nursing instructions specific to steroid pulse therapy may improve the interaction between nurses and patients, thus enhancing nursing care and patient understanding, ultimately achieving positive behavior modification. Many studies have illustrated how nursing instruction positively affects patient attitudes, knowledge, and ultimately disease course and care [12-16]. In addition, good nursing instruction may reduce the incidence of complications and hospital stay, thus leading to economic benefits [17].

Patient needs differ based on the influence of clinical symptoms. For example, patients receiving chemotherapy have physical discomfort (nausea, vomiting, fatigue, poor appetite) and psychological symptoms (anxiety, depression) and hygiene is a concern, thus creating unique nursing demands [10]. Burn patients have also been shown to have unique needs and concerns, thus, influencing the degree and type of nursing instruction that is required [18].

Orem's Theory of Self-Care is based on the philosophy that "all patients wish to care for themselves" and they can recover more quickly and holistically if they are allowed to perform their own self-care to the best of their ability. Nurses can provide a complete healthcare system, a partial healthcare system, or a supportive education system.

Our results indicate that patients undergoing steroid pulse therapy are in need of nursing instruction in all categories (except optic) at the 4 time points examined (before therapy, after therapy, one week after therapy, and two weeks after therapy), and that nursing instruction improved patients' knowledge level. Prior study has indicated that the need of nursing instructions was lower in patients receiving steroid pulse therapy [19]; therefore, booklets given to provide nursing instruction were those designed for chronic renal failure patients. Chen *et al. *[20] pointed out that nursing instruction can increase a patient's health care knowledge and may help to reduce the incidence of unplanned return visits. In studies of women with breast cancer, individual nursing instruction was shown to help patients develop and implement a better plan for their overall health and lifestyle, and to reduce uncertainty about their disease [21,22].

In our study, we found that patients with a significant increase in body weight required nursing instruction based on our category endocrine system. This is likely due to sodium and water retention as a result of the high steroid dose. With a significant weight change, patients develop self-image concerns. Permut [23] indicates that patients should be made aware of the side-effects caused by drug therapy, such as body weight increase, menstrual changes, and muscular weakness, as well as an increased chance of infection [24].

We also found that the need of nursing instruction was related to pain level. Jensen's knowledge-behavioral model of pain predicts that belief influences behavioral function [25]. In other words, a patient's behavior and their knowledge function may be affected by the experience of pain. Thus, if nurses can provide a positive concept of pain during therapy, it may help to reduce a patient's experience of pain.

Our results showed that length of admission was statistically correlated with symptoms related to the gastrointestinal and nervous systems. Patients who receive steroid pulse therapy usually have increased gastric acid, abdominal distension, hyperactivity, and insomnia. Informing patients that these symptoms are only temporary, and providing related nursing instruction for symptom relief may assist in reducing a patient's physical discomfort and anxiety. Reducing a patient's physical discomfort and anxiety could potentially lead to a reduction of overall length of hospital stay.

Knowledge can be defined as one's understanding and attitude toward something or someone. Turkington *et al. *[26] describes knowledge with emphasis on the intrinsic attitude, belief, and manner one thinks about something, and how it influences emotion and behavior. Our results indicated that the knowledge level of patients receiving pulse therapy was significantly higher after nursing instruction. Other studies have also reported that the knowledge level of subjects with chronic disease is improved with nursing instruction [27]. Nursing instruction discussing preventive medicine in more detail with patients provides an efficient way to increase disease prevention and further enhance patient knowledge of disease symptoms [28].

In our study of the predictors of the need for nursing instruction, 30.1% of the variability was explained by body weight change and knowledge level. Based on the clinical observation, patients who received steroid pulse therapy usually experienced a temporary increase in body weight due to the retention of water after treatment. Thus, it is important for nurses to explain the reasons for the increase in body weight in detail. Study has shown knowledge level and nursing instruction are predictors of influence and patient outcome [29].

This study possesses limitations that should be considered. Because all patients were from a single Taiwanese medical center, the results may not be generalizable to all steroid pulse therapy patients. Another major limitation is that the study was not a randomized control trial. Subjects were recruited in 6 months. Therefore we do not know whether patients would have gained his knowledge during standard nursing care before entering the investigation. Furthermore, patients could not be divided into control and nursing instruction groups, because all patients should receive appropriate nursing instructions. To not provide all patients with the best instruction possible would not be ethical. Finally, some subjects were lost to follow-up, and this may have introduced bias into the results. However, 90% of the subjects finished the study, thus we believe that the results were not significantly affected by the number lost to follow-up.

## Conclusions

Steroid pulse therapy patients are in need of nursing instruction at four different time points, and patient knowledge level is increased with appropriate nursing instruction. Nurses should design self-care instructions specific for patients undergoing steroid pulse therapy according to the priority of patient symptoms to help improve patient perceptions related to disease and treatments and achieve the goals of self-care and health maintenance.

## Competing interests

The authors declare that they have no competing interests.

## Pre-publication history

The pre-publication history for this paper can be accessed here:

http://www.biomedcentral.com/1471-2474/11/217/prepub
